# Using social annotation to construct knowledge with others: A case study across undergraduate courses

**DOI:** 10.12688/f1000research.109525.1

**Published:** 2022-02-25

**Authors:** Esteban Morales, Jeremiah H. Kalir, Alice Fleerackers, Juan Pablo Alperin

**Affiliations:** 1 Faculty of Education, University of British Columbia, Vancouver, BC, V6T 1Z2, Canada; 2 Scholarly Communications Lab, Simon Fraser University, Vancouver, BC, V6B 5K3, Canada; 3 School of Education and Human Development, University of Colorado Denver, Denver, Colorado, 80204, USA; 4 Interdisciplinary Studies, Simon Fraser University, Vancouver, BC, V6B 5K3, Canada; 5 School of Publishing, Simon Fraser University, Vancouver, BC, V6B 5K3, Canada

**Keywords:** social annotation, knowledge construction, computer-supported collaborative learning, case study

## Abstract

**Background:** Social annotation (SA) is a genre of learning technology that enables the addition of digital notes to shared texts and affords contextualized peer-to-peer online discussion. A small body of literature examines how SA, as asynchronous online discussion, can contribute to students’ knowledge construction (KC)—or a process whereby learners collaborate through shared socio-cognitive practices. This case study analyzed how SA enabled student participation in seven KC activities, such as interpretation and elaboration.

**Methods:** We analyzed 2,121 annotations written by 59 students in three undergraduate courses at a Canadian University in the Winter 2019 semester. Using a method of open coding and constant comparison, we coded each annotation for evidence of KC activities.

**Results:** Results showed a range of KC activities in students’ SA. Across courses, interpretation was the most common KC activity (40%), followed by elaboration (20%). Annotations that were part of peer-to-peer discussion included all seven types of KC activities, but some activities, such as consensus building, support, and conflict, were almost exclusively found in replies to others.

**Conclusions:** This study suggests that SA is a productive form of online learning through which undergraduate students in multiple disciplinary contexts can interact with peers, make sense of academic content, and construct knowledge by reading and writing together.

## Introduction

Online conversation—as with chat rooms or discussion forums—is a ubiquitous practice with societal implications ( Paulus and Wise 2019). In educational contexts, online discussion emerged alongside popular online bulletin boards in the mid-1990s, and written digital discourse is now an essential feature of contemporary educational technologies, such as learning management systems (LMS), discussion boards, and blogs ( Weller 2020). Online discussion allows students to converse about topics pertinent to course content ( Loncar, Barrett, and Liu 2014), is often asynchronous ( Sheail 2018), and encourages learners’ participation at their own pace. Asynchronous online discussion is a tenet of social learning in digital environments ( Hill, Song, and West 2009) with research indicating such discourse enables social and collaborative learning ( Chan and Pow 2020; Hambacher, Ginn, and Slater 2018; McMahon 1997).

The growth of asynchronous online discussion in digital education presents both opportunities and challenges. For learners, the benefits of online discussion include social knowledge construction (KC) ( Eryilmaz *et al*. 2013; Kent, Laslo, and Rafaeli 2016), meaningful dialogue with information shared and negotiated ( Gao, Zhang, and Franklin 2013; Wise, Hausknecht, and Zhao 2014), collaboration with peers ( Pratt and Back 2013), and reflection ( Truhlar, Walter, and Williams 2018). Online discussion can improve student learning processes and outcomes ( Hambacher, Ginn, and Slater 2018; Kent, Laslo, and Rafaeli 2016). Alternatively, online discussion has been associated with low levels of student participation ( Aloni and Harrington 2018), instructor bias ( Baker *et al*. 2018), and non-substantive learner interaction ( Hambacher, Ginn, and Slater 2018), perhaps due to the imposition of discussion structure and order ( Gao, Zhang, and Franklin 2013). Indeed, the “dreaded threaded” discussion has been a trope of unsatisfying and unproductive online learning for at least two decades ( Chabon, Cain, and Lee-Wilkerson 2001). The respective benefits and challenges of online discussion motivates additional research on how text-based, asynchronous online discourse can productively promote learners’ interaction, collaboration, and reflection in digital learning environments.

Given the ubiquity and timeliness of asynchronous online discussion, particularly in higher education ( Bettinger *et al*. 2017), our study examines undergraduate student participation in social annotation (SA) as a form of online discussion. Specifically, we studied how peer-to-peer dialogue via SA contributed to KC activities in multiple courses from different disciplines. We first reviewed literature about SA and KC, highlighting the relevance of this relationship to online discussion and learning. We then present a case study of KC activities and patterns in 2,121 annotations written by students from three undergraduate courses at a Canadian university. We present findings about: a) The discursive, or peer-to-peer, characteristics of student SA; b) the prevalence of KC activities evidenced in student SA; and c) patterns of KC activities in student SA, including a comparison among courses. Our discussion considers the strengths and limitations of this study, the social qualities and value of student participation in SA, and implications for the use of SA as asynchronous online discussion.

## SA, collaboration, and KC

In this study, we embrace a sociocultural stance toward learning ( Gutiérrez and Rogoff 2003; John-Steiner and Holdbrook 1996). We extend computer-supported collaborative learning (CSCL) research that considers cognition a socially-situated, group accomplishment ( Enyedy and Hoadley 2006; Stahl 2017). We recognize communication, whether spoken or written, as central to the social construction of reality ( McMahon 1997); consequently, social and discursive activities like thinking aloud, asking questions, and providing explanation promote meaningful learning ( King 2007). We understand collaboration as a “social contract” ( Dillenbourg 1999) reflecting shared situations and interactions, as well as orchestrated participation in online learning (i.e., Chen *et al*. 2018). Our orientation to cognition, communication, and collaboration positions us to study how SA as online discussion can encourage engagement in structured dialogue and shared epistemic practices ( Kalir 2020a; Eryilmaz *et al*. 2013).

### Social annotation

Annotation—or the addition of notes to texts ( Kalir and Garcia 2021)—has, for centuries, informed how people read, write, and interact with texts and other readers ( Jackson 2001). Today, the proliferation of digital annotation tools ( Wolfe and Neuwirth 2001), particularly in education as with SA technology (i.e., Paradis and Fendt 2016; Seatter 2019; Zhu *et al*. 2020), has enabled readers to annotate online documents using text, links, and multimedia while engaging in dialogue. SA affords contextualized discussion as peer-to-peer dialogue is “anchored” to a source text ( Gao, Zhang, and Franklin 2013), in contrast to conventional online discussion forums, which are distal from learners’ texts. Anchored online discussion helps learners acquire discipline-specific terminology and methods ( Kararo and McCartney 2019), collaborate with peers ( Chan and Pow 2020), and engage in public discourse ( Kalir and Garcia 2019; Marshall and Brush 2004). SA is an alternative approach to online discussion forums as anchored dialogue enables learners to engage in proximal, meaningful conversation with texts and peers ( Plevinski, Weible, and Deschryver 2017).

In higher education—the context of our study—a growing body of research indicates that SA promotes productive online discussion and student learning. Novak *et al*. (2012) reviewed SA use across seven higher education disciplines and found that student reading comprehension, peer review, motivation, and attitudes toward technology use were all positively influenced by SA activities. A more recent systematic review, conducted by Zhu and colleagues (2020), highlighted how SA can help students process domain-specific knowledge, support argumentation and inquiry, improve literacy skills, and can aid instructor and peer assessment. As online discussion, SA promotes critical thinking via peer-to-peer dialogue ( Mendenhall and Johnson 2010), builds collaborative sensemaking ( Chen 2019), and can offer students social, linguistic, and cultural learning opportunities (i.e., Brown and Croft 2020; Thoms and Poole 2017). Because SA affords dialogic, collaborative learning in digital learning environments (i.e., Allred, Hochstetler, and Goering 2020; Sprouse 2018; Wranovix and Isbell 2020), it is appropriate to further investigate how SA—as asynchronous online discussion—supports peer-to-peer activity like KC.

### Knowledge construction

With roots in cognitive psychology and constructivism, KC is defined in the CSCL literature as a social process “by which students solve problems and construct understanding of concepts, phenomena, and situations” ( van Aalst 2009, 261). In online learning, KC emphasizes the social processes whereby divergence of ideas lead to the convergence of negotiated meanings ( Onrubia and Engel 2009). KC differs conceptually and processually from the transmission of information (knowledge sharing) or the innovative use of ideas and tools (knowledge creation). KC relies on peer-to-peer dialogue as an instrument for learning ( Pena-Shaff and Nicholls 2004), group participation in shared problem-solving environments and opportunities ( Hmelo-Silver and Chernobilsky 2004) and concerns how different perspectives are assimilated among groups and incorporated into individual thinking and metacognition ( Yu and Wu 2016; Luo and Clifton 2017). KC activities—such as learners’ collaborative engagement in elaboration, argumentation, question-asking, and explanation (i.e., Fu, van Aalst, and Chan 2016)—are understood as situated, reflexive, and related to deep learning (De Wever *et al*. 2009; van Aalst 2009).

Not every online interaction among students leads to KC or learning. Nonetheless, CSCL research has shown that learners’ technology-mediated, dialogic interaction (i.e., Enyedy and Hoadley 2006; Hambacher *et al*. 2018) can lead to the meaningful co-construction of knowledge ( Heo, Lim, and Kim 2010) and collaborative learning ( Eryilmaz *et al*. 2013). KC activities are frequently associated with student digital dialogue as such conversation has the potential to “increase the level of participation and interaction among students and … has the capacity to provide a meaningful supplement to regular class discussions” ( Pena-Shaff and Nicholls 2004, 264). Online discussion presents an ideal scenario within which to research KC as students’ interaction patterns may be easily orchestrated ( Hmelo-Silver and Chernobilsky 2004), accessed ( Schrire 2006), and analyzed to differentiate among cognitive tasks and accomplishments ( Luo and Clifton 2017). For example, when a group of students discuss project management, they may engage in socio-cognitive KC processes like questioning, summarizing, and elaborating ( Onrubia and Engel 2009). Whereas most studies of student KC in the CSCL literature examine activity in more conventional discussion forums, SA may also productively promote KC activities through anchored discourse that encourages collaborative and meaningful learner dialogue ( van der Pol, Admiraal, and Simons 2006).

### SA enabling KC

There are but a handful of studies that examine how SA, as online discussion, can enable KC activities. Eryilmaz et al. (2013) describe how SA reduced coordination activities among learners, which consequently lead to greater individual learning gains and increased some group KC activities like elaboration and conflict. Plevinski, Weible, and Deschryver (2017) found that SA can support multiple KC activities, primarily learner elaboration and interpretation, determining that “coordination activities were relatively minimal and that [anchored annotation systems] supported KC activities closely aligned with the cognitive processes of remembering and understanding” (117). Zarzour and Sellami (2017) concluded that SA effectively encouraged learner engagement with multiple perspectives, with students “gaining ideas, seeing others’ different viewpoints, linking more external data, and building knowledge about the annotated content” (394). Kalir (2020b) detailed how SA discussion supported a repertoire of group-level epistemic expressions including critical inquiry, associative connections across contexts, and discernment among multiple perspectives.

Our study is motivated by opportunities for further scholarly inquiry at the intersection of KC and SA. There is little discussion in the literature about how different SA practices lead to specific KC activities and patterns of students’ social interaction. There is also a need to further understand how KC promoted by SA may vary across instructional settings and disciplinary contexts. Our case study therefore focuses on social practices afforded by SA as online discussion and analyzes how SA enables student participation in KC activities. We do so by studying student SA in three undergraduate courses at a Canadian university. Specifically, our study addresses three research questions (RQs):

(1) How does student SA and the prevalence of discursive threads differ across courses from multiple disciplines?
(2) What specific KC activities are most frequently observed within student SA, and how do KC activities differ across courses?
(3) What patterns of KC activities are most frequently observed within students’ discursive SA, and how do patterns of discursive KC activities differ across courses?

## Methods

This study was conducted using data collected for the assessment of students as part of their regular academic work and, as such, was exempt from ethics board review according to Article 2.5 of the Canadian Tri-Council Policy Statement: Ethical Conduct for Research Involving Humans.

This research employed a single-case study design ( Yin 2009) at a Canadian university throughout the winter 2019 semester. Seven faculty from multiple disciplines at the undergraduate and graduate levels were invited to integrate SA as online discussion into their courses using Hypothesis (Hypothes.is, RRID:SCR_000430) ( https://web.hypothes.is/), an open-source technology that enables SA and which has been widely studied in higher education (e.g., Chen 2019; Goller *et al*. 2021; Sprouse 2018; Seatter 2019). Faculty were provided with technical and pedagogical support by the research team.

All seven courses used SA for asynchronous online discussion in some capacity. Three were selected for this study: Publishing Studies (PUB); Gerontology (GERO); and Gender, Sexuality and Women’s Studies (GSWS). These courses were selected using the following inclusion criteria to assure the quality and consistency of data: 1) Similar course-level (upper-level undergraduate); 2) High student engagement with SA as evidenced by number of annotations; and 3) Close contact between the instructor of each course and the research team.

As detailed in our related research about student perceptions of SA ( Kalir *et al*. 2020), these three courses differed in terms of instructor pedagogy, enrolled students, and the use of SA for online discussion. PUB organized class sessions into two components—a lecture followed by face-to-face discussion—and required that students make at least two annotations on each reading before class to receive a 15% participation grade. GERO sessions typically started with student face-to-face discussion and then transitioned to a traditional lecture. SA represented 15% of students’ final grades and was assessed using a rubric that included engagement with peers and annotation consistency, quantity, and originality. GSWS sessions relied on a combination of lectures and seminars; participation accounted for 20% of students’ final grade and included SA. Table 1 summarizes student engagement with SA in the three courses.

**Table 1.** Summary of courses, participating students, and annotation activity.

Course	Number of students	Number of annotators	Total annotations	Mean annotation length (Characters)
PUB	26	26	1,118	274.1
GERO	8	8	759	158.6
GSWS	25	22	267	192.6

PUB, Publishing Studies; GERO, Gerontology; GSWS, Gender, Sexuality and Women’s Studies.

### Data collection and analysis

A total of 59 students from PUB, GERO, and GSWS authored 2,121 annotations during online discussion (instructors’ 24 annotations were removed from analysis). When the term ended, SA was collected using the Hypothesis API to provide detail of annotation data and metadata. This enabled our team to describe basic characteristics of student SA—including descriptive statistics of total annotations and replies, discursive threads per course, and SA mean length—as well as to analyze student annotation content for evidence of KC activities.

The content of student SA was analyzed using open coding and constant comparison ( Strauss and Corbin 1990), with all 2,121 annotations coded for evidence of KC. A single annotation could be coded as exhibiting more than one KC activity. To do so, we adapted the codebook developed by Plevinski, Weible and Deschryver (2017) which draws on relevant CSCL literature to identify seven KC activities: Clarification, conflict, consensus building, elaboration, interpretation, question, and support. Definitions and examples for these codes are provided in Table 2 ( Morales, Fleerackers, Alperin 2022).

**Table 2.** Codebook used to analyze knowledge construction activities. Numbers in square brackets (e.g. [SA100]) represent anonymized identification for each annotation.

Code	Definition	Example SA
Clarification	Listing main ideas, facts from the reading, assumptions	“Ethnicity is both collective/external (social interaction) and individual/internal (personal identification)” [SA722]
Conflict	Disagreeing with another student; mentioning a different point of view in direct reply	“I agree, but i also think health services has been gradually improving since then (citation was referenced almost a decade ago)” [SA308]
Consensus building	Discussion of misunderstandings; reaching an agreement on an idea, fact, or interpretation; negotiating the definition, interpretation, or truthfulness of a claim or fact.	“I agree with you, selective system. The policy makers only favour "some" people. The policy heavily rest on familiar function and policy makers are reluctant to provide resources to older immigrants.” [SA334]
Elaboration	Connecting ideas with examples, defining terms, causes or consequences, listing advantages or disadvantages, using analogies to explore ideas, making connections, comparing and contrasting	“This makes me think of the term ‘wokenomics’; the current trend of brands using hot button social and political issues to sell products” [SA745]
Interpretation	Inferences, conclusions, summaries, generalizations, problem solution suggestions, hypotheses	“Perhaps some relatively special topics can only be initiated by small publishers, and large publishers may only focus on issues that the public is aware of, and they have reasons for doing so” [SA132]
Question	Seeking to find additional information pertaining to the discussion; prompt further discussion about the current topic; a question that reflects upon the current discussion	“I wonder if the term ‘abuse’ in itself scares away individuals (young and old) to report their treatment and to seek support” [SA409]
Support	Empathizing; statements of acknowledgement; providing direct feedback	“This is a good point to bring up… we do not want the health professionals to put their own views of what types of physical activities a person should do (…)” [SA164]

To assess the reliability of Plevinski *et al*.’ (2017) codebook in the context of our multi-course analysis, two authors (EM and AF) independently coded a set of student annotations from all three courses and compared results. The authors’ coding was measured and achieved a Kappa value of 0.86, which is considered to be a strong level of rater agreement ( Mchugh 2012). EM subsequently coded the remaining annotations for evidence of KC activities. The coding process used NVivo version 12 (NVivo, RRID:SCR_014802) ( http://www.qsrinternational.com/nvivo-product) (freely available alternative software: Taguette, https://www.taguette.org/). Annotations that lacked evidence of KC activity were often associated with coordination, such as “This relates to what I highlighted earlier in the paper” [631], or with informal social interactions, like “Hello world!” [739].

## Results

Our study examined the use of SA for online discussion in three different undergraduate courses and features three complementary sets of findings. To address RQ1, we report characteristics associated with student SA by categorizing the types of annotation written by students and identifying the prevalence of discursive—or peer-to-peer—threads composed via SA. In response to RQ2, we present quantitative summaries of KC activities evidenced in student SA. A third set of findings addressing RQ3 details the patterns of KC activities most frequently observed within students’ SA and identifies how patterns of KC activities within discursive threads differed across courses.

### Characteristics of discursive SA

**Figure 1.**
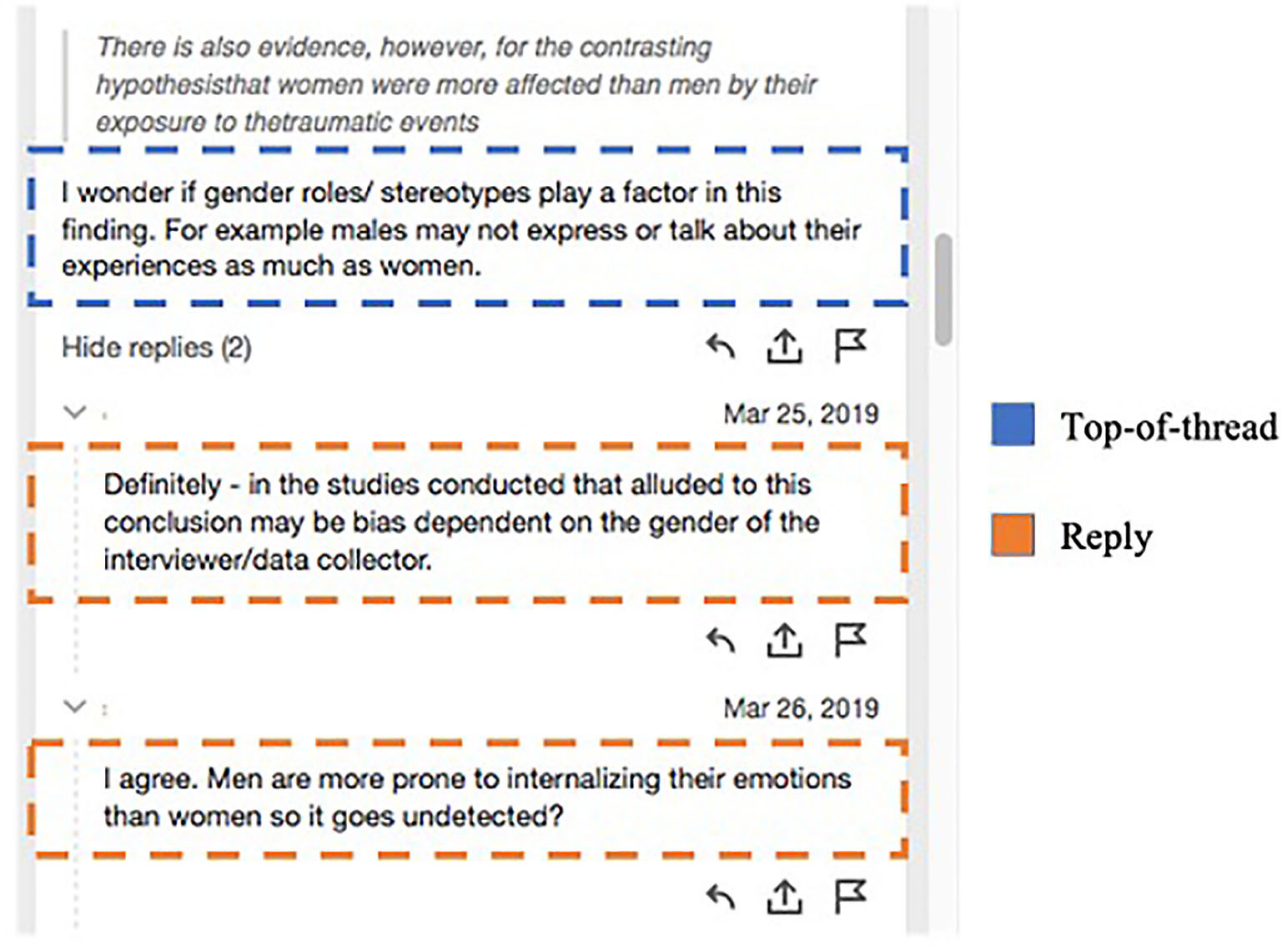
Sample SA thread (student names removed) with one top-of-thread annotation and two replies. SA, social annotation.

We first report three types of annotation written by students to identify the basic discursive characteristics of SA for online discussion. This first set of findings distinguishes individual annotations from those that were part of discursive threads. A thread is an instance in which one students’ annotation elicited at least one reply from a peer ( Figure 1). As reported in Table 3, all student SAs were categorized as: a) an individual annotation that did not appear in a thread and elicited no peer response, henceforth referred to as *no-thread*; b) an annotation that elicited at least one peer response, referred to as *top-of-thread*; and c) an annotation reply that developed discursive interaction among peers. In reporting our findings, it is important to recall that even no-thread SA had social qualities; this category of annotation was written for group-level participation in each course and was visible to peers throughout online discussion activities.

**Table 3.** Categories of student SA by course.

Course	No-thread	Top-of-thread	Reply	Total
GERO	402	135	212	749
GSWS	161	37	56	254
PUB	1,056	30	32	1,118
Total	1,619	202	300	2,121

SA, social annotation; PUB, Publishing Studies; GERO, Gerontology; GSWS, Gender, Sexuality and Women’s Studies.

Across all three courses, 76% (1,619 annotations) of student SA received no peer response and were not part of any threaded discussion (no-thread). A total of 24% (502) of all SA appeared in a thread, with 10% (202) of annotations as top-of-thread and 14% (300) as a reply. In GERO, nearly half (46%) of student SA appeared in threads, including 135 top-of-thread annotations and 212 replies. In GSWS, just over one third (36%) of student SA appeared in threads, with 37 top-of-thread annotations and 56 replies. And in PUB, only 6% of student SA were in threads, with 30 top-of-thread annotations and 32 replies.

### Frequency of KC across courses

Whether or not student SA appeared in a discursive thread, we found that 71% (1,511) of all annotations included a single KC activity, 18% (380) included two KC activities, and 5% (105) included three or more KC activities. A total of 6% of all annotations (125) did not include evidence of any KC activity. Across courses, the most common KC activity was interpretation: 40% of all annotations (1,051) included interpretation in the form of an inference, conclusion, or summary of the text. Examples of interpretation included a GERO student who wrote “[i] t seems that this is a big problem with categorizing but is it possible to avoid ‘othering’? to me, it seems we can not avoid it in research because that is how we can compare and contrast findings to present conclusions” [SA725], as well as a GSWS student who observed, “I think this is a really great point for illustrating how media coverage isn’t just about representation, but about a lack of representation” [SA050].

**Figure 2.**
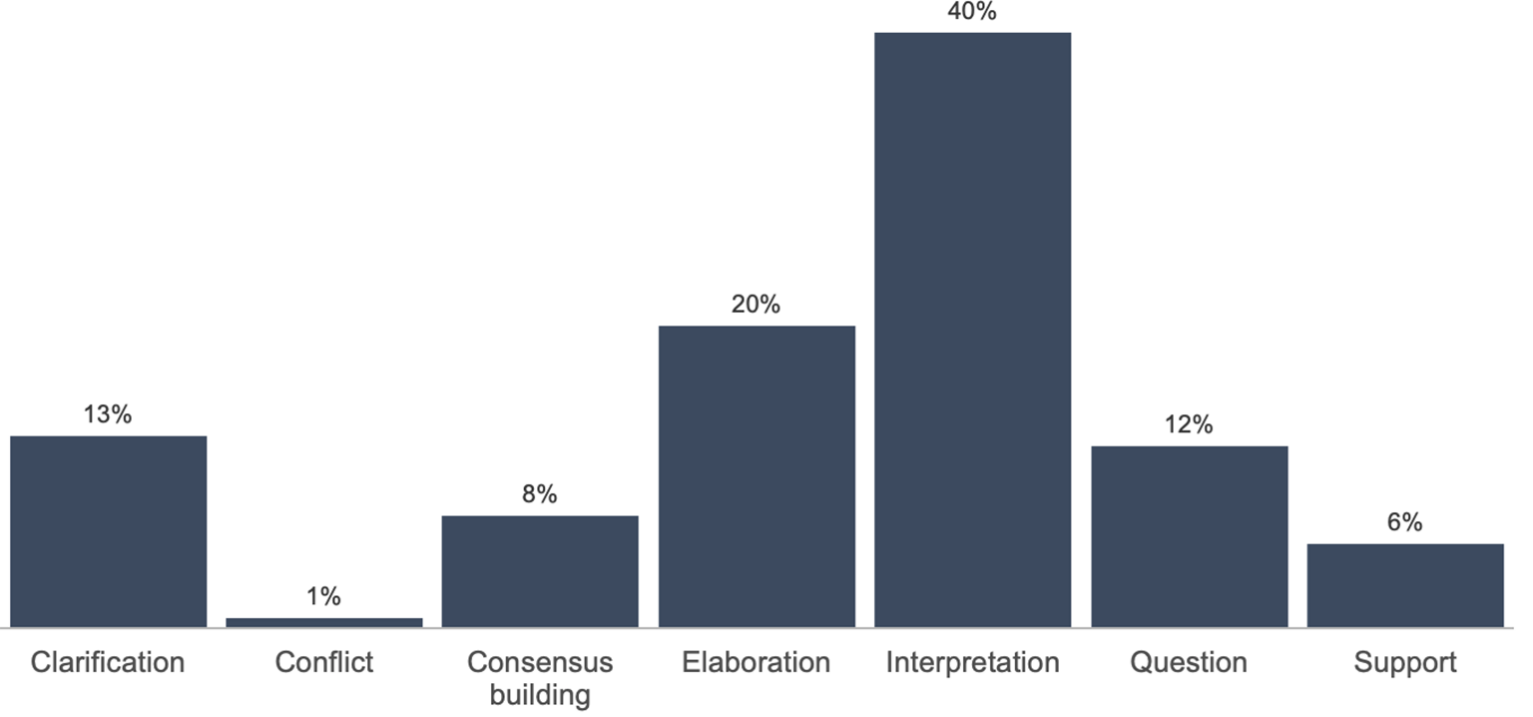
Aggregated distribution of KC activities across courses. KC, knowledge construction.

As summarized in Figure 2 ( Morales, Fleerackers, Alperin 2022), elaboration was the second most common KC activity featured in all student SA, appearing in 20% of all annotations (532). For example, a student in PUB wrote “[t] his is similar to what we see with the Internet and the use of tools like Facebook or Twitter or blogging. The Internet is fairly accessible to a large percentage of the world where anyone can post anything they want to” [SA917]. Across our study sample, other KC activities present in students’ SA included clarification (13% of all annotations), asking a question (12%), consensus building (8%) and providing support (6%). Conflict was the least common KC activity across courses and occurred in less than 1% of annotations, indicating that students may have avoided contrasting points of views when writing SA for online discussion.

**Figure 3.**
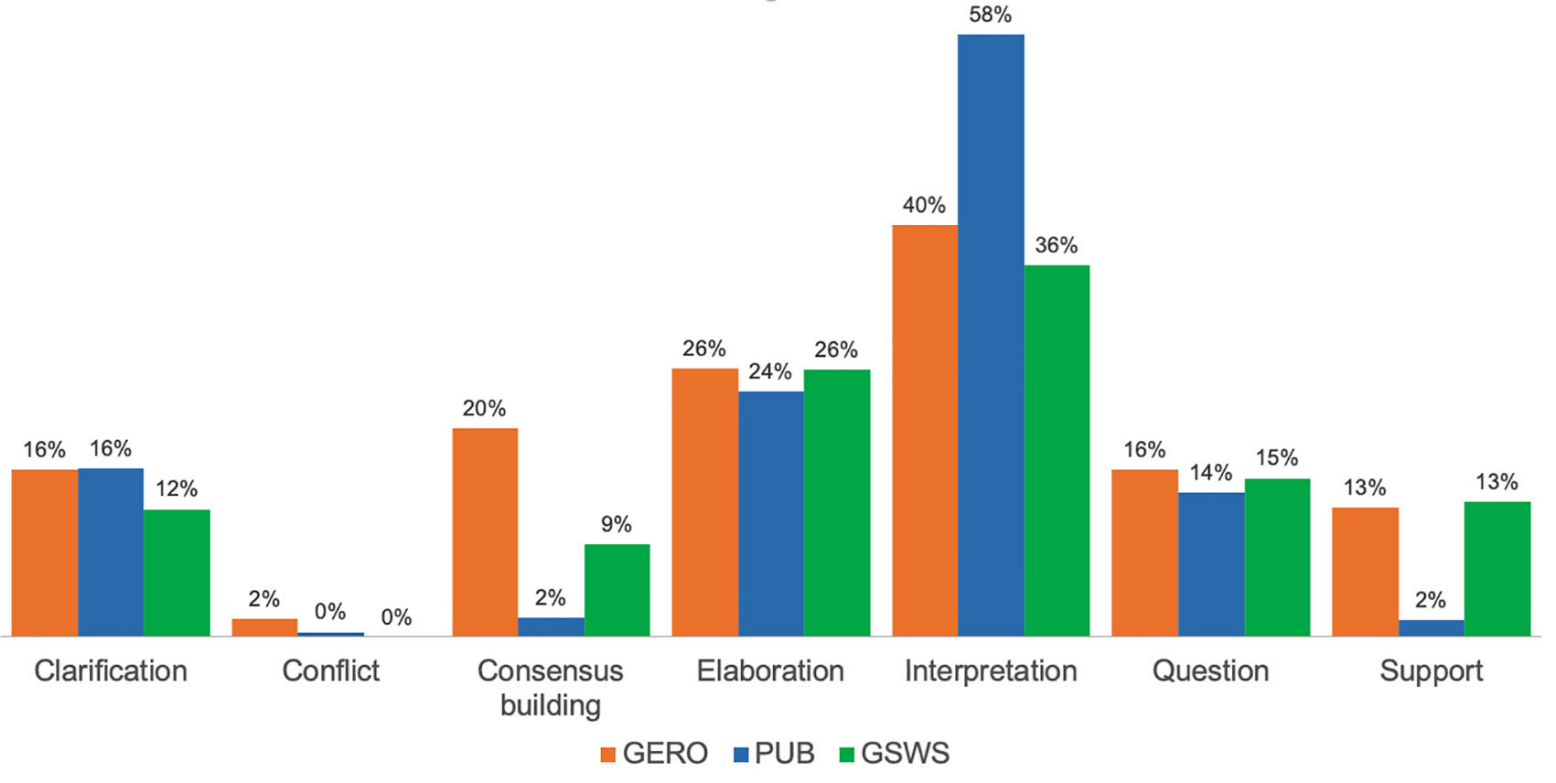
Distribution of KC activities per course. KC, knowledge construction; PUB, Publishing Studies; GERO, Gerontology; GSWS, Gender, Sexuality and Women’s Studies.

The distribution of KC activities within online discussion further demonstrated the prevalence of interpretation and elaboration in student SA. As indicated in Figure 3, interpretation accounted for 58% of the KC activities within PUB and over one third of KC activities in GERO and GSWS. Elaboration occurred in 26% of the KC activities of GERO and GSWS, and 24% of KC activities in PUB. The prevalence of other KC activities differed among the three courses. Students in GERO, for example, more frequently included consensus building in their SA during online discussion (“I agree, this question is very subjective but I wonder if that is the point for this study” [SA293]), whereas few annotations from PUB included consensus building or support. Results further suggest that the KC activities of asking a question and providing clarification appeared in students’ online discussion with about the same frequency across all three courses.

### KC patterns among discursive SA

**Figure 4.**
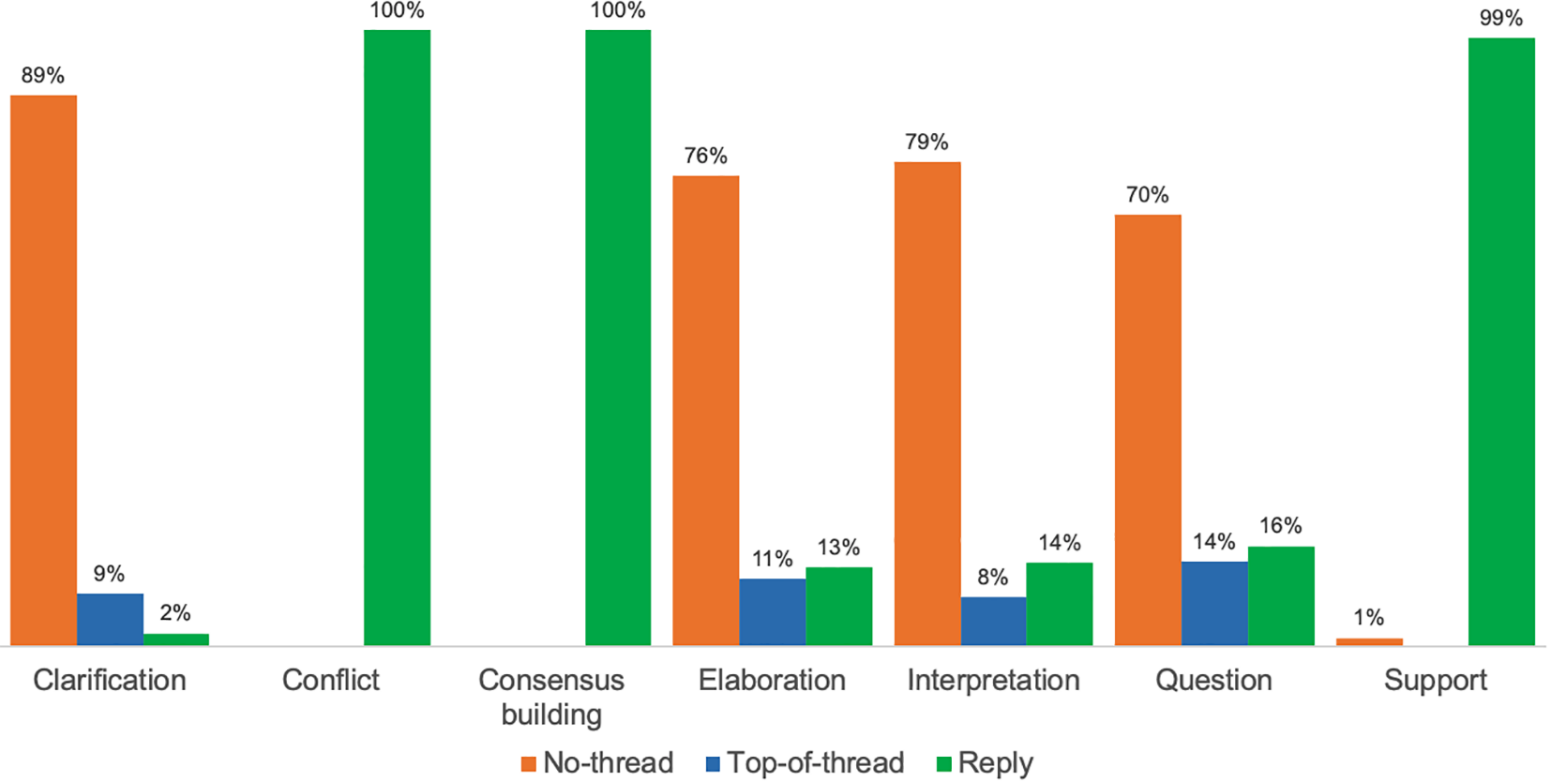
Distribution of KC activities per SA category. KC, knowledge construction; SA, social annotation.

Our third set of findings detail patterns of KC activities within discursive, or peer-to-peer, SA and includes a comparison of differences among courses. To address RQ3, we first recall that over three-quarters of student SA in this study (76%) received no peer response and were not part of threaded discussion (no-thread annotation). Figure 4 illustrates the distribution and prevalence of seven KC activities (as detailed in RQ2) by SA category (as described in RQ1).

Across courses, four KC activities were predominantly concentrated in annotations that were not part of a thread: Interpretation (with 79% of the 1,051 annotations evidencing interpretation found in no-thread annotations); elaboration (76% of 532 annotations); clarification (89% of 338 annotations); and asking a question (70% of 320 annotations). Among these four most prominent KC activities, less than one-third of student SA that included a question was discursive, only about one-quarter evidencing interpretation or elaboration was discursive, and just one in ten annotations that provided clarification were discursive. In PUB, for example, in which 94% of student SAs were not in threads, prominent KC activities were interpretation (52% of 1,056 no-thread annotations), elaboration (21%), clarification (15%), and asking a question (12%). In GERO, the course with the lowest percentage of no-thread annotation (54%), prominent KC activities within this category included interpretation (36% of 402 no-thread annotations), elaboration (26%), and clarification (25%). Similarly, in GSWS—in which approximately two-thirds of student SA were no-thread—the most frequent KC activities in this subset were interpretation (40% of 161 no-thread annotations) and elaboration (29%).

Students’ KC activities also occurred in the 24% of SA that were discursive and located within threads as either top-of-thread or a reply. Four of the same five KC activities found among no-thread annotation were also identified, across all three courses, and with approximately the same frequency, in annotations that began threads: Interpretation, in 8% of 1,051 annotations evidencing interpretation; elaboration (11% of 532 annotations); clarification (9% of 338 annotations); and asking a question (14% of 320 annotations).

As previously shown in Figure 4, all seven KC activities were found in students’ replies to peers. Three KC activities were almost exclusively discursive, appearing only in student replies: consensus building (with 100% of the 198 annotations coded for consensus occurring in replies); support (99% of 148 annotations); and conflict (100% of 18 annotations). Some KC activities were infrequently evident in replies, such as clarification (2% of 338 annotations).

A further analysis of peer-to-peer online discussion in GERO, GSWS, and PUB reveals course-level patterns of KC activity in students’ discursive SA. Table 4 reports the percentage of annotations that contained evidence of each KC activity among annotations in threads—either as top-of-thread or as a reply—for each of the three courses. In both GERO and GSWS, prominent KC activities that appeared in top-of-thread SA included interpretation and elaboration. Interpretation was also the most common KC activity among top-of-thread SA in PUB. Across courses, interpretation, consensus building, and support were the three KC activities that more frequently appeared in replies. Overall, the three courses showed relatively similar patterns of KC activities among SA threads.

**Table 4.** Percentage, by course, of discursive SA with evidence of KC activities.

Course	SA Category	Clarification	Conflict	Consensus building	Elaboration	Interpretation	Question	Support
GERO	Top-of-thread	3%	0%	0%	6%	8%	6%	0%
	Reply	0%	2%	26%	9%	18%	6%	16%
GSWS	Top-of-thread	5%	0%	0%	9%	10%	4%	0%
	Reply	1%	0%	18%	7%	13%	7%	26%
PUB	Top-of-thread	3%	0%	0%	7%	20%	4%	0%
	Reply	3%	4%	19%	4%	16%	4%	16%

SA, social annotation; KC, knowledge construction; PUB, Publishing Studies; GERO, Gerontology; GSWS, Gender, Sexuality and Women’s Studies.

Two examples illustrate course-level patterns of KC present in students’ discursive SA, specifically interpretation and elaboration appearing atop threads followed by consensus building and additional interpretation in replies. One GERO student’s top-of-thread annotation (coded for both interpretation and elaboration) noted: “I was surprised that so many people disagree with improving the quality of life for immigrant seniors. The commenters are ‘othering’ and blaming the immigrants for not adapting to ‘Canadian culture.’ It is a sad reality that people believe this, especially since Canada was colonized by immigrants” [SA624]. A peer’s reply included consensus building and interpretation: “Canadian culture is very diverse - agreed! I feel this is also the reason that everyone needs to compromise to come up with a sustainable solution for all because it’s near impossible to cater to all the specific/individualized needs of such a diverse society” [SA587]. In PUB, one top-of-thread annotation that demonstrated interpretation stated: “Mentors and role models for diversity are so important in the workplace. When people trying to break into the publishing world see others succeeding that they can identify with, they may be inspired and more confident to chase after their goals” [SA274]. In response, a peer annotation featured consensus building and interpretation: “I agree. To add to your point, connections are so important in the job market now. To get into an industry, it seems like you need to know someone. If the industry is dominated by white people, and those people only are connected to other white people, then it would be hard for people of other backgrounds to get a foot in the door” [SA171].

## Discussion

This descriptive study of three undergraduate courses from different disciplines at one university examined how student participation in asynchronous online discussion via SA contributed to KC activities. Our study builds on research about online discussion in higher education (i.e., Chen *et al*. 2018; Sun and Gao 2017) and extends insight from CSCL literature regarding the role of social learning technologies to enable learner cognition, communication, and collaboration (i.e., Chan and Pow 2020; King 2007). From this stance, SA—as a popular approach to online discussion ( Allred, Hochstetler, and Goering 2020; Zhu *et al*. 2020)—was studied because it allowed students to add interactive notes to shared digital texts, anchor discussion in meaningful social contexts ( Gao, Zhang, and Franklin 2013), and make their thinking visible and responsive to peers ( Kalir and Garcia 2019; Marshall and Brush 2004). It has been nearly a decade since Novak et al. (2012) encouraged investigation about the promises and limitations of SA across varied higher education learning environments and among diverse groups of learners. While subsequent research has documented SA as productively mediating collaborative dialogue and learning ( Brown and Croft 2020; Wranovix and Isbell 2020), only a small subset of CSCL literature details the intersection of SA practices and KC activities. In this discussion we: Address the contributions of this exploratory case study, with attention to the strengths and limitations of our research design and context; consider the discursive qualities and social value of student SA as participation in KC activities; and present methodological and instructional implications for SA as asynchronous online discussion.

The design of our study included multiple features that strengthened the relevance of this case for researchers interested in online discussion and CSCL constructs like KC. First, we expanded on a small but important literature that examines KC activities as enabled by SA ( Eryilmaz *et al*. 2013; Plevinski *et al*. 2017; Zarzour and Sellami 2017). Whereas previous studies examined student KC within a single discipline, our case is the first instance to document undergraduate students’ KC via SA across three disciplines and does so with an SA corpus larger than that of prior studies. Second, we studied KC activities made visible by the SA technology Hypothesis, which strengthens our study by making use of a SA technology that has been both widely studied and widely adopted by global educational institutions ( https://web.hypothes.is/blog/our-view-from-20-million-annotations/). Third, our approach to data analysis borrowed from Plevinski *et al.’* (2017) codebook to deductively identify seven KC activities in student SA. We hope our findings further establish these particular activities and definitions as the future benchmark when investigating SA for evidence of KC. A fourth strength of this case is its comprehensive account of students’ collaborative learning as aided by SA. Having previously reported how this sample of students perceived SA as a valuable contribution to their learning ( Kalir *et al*. 2020), we can now pair prior insight with these findings about student participation in SA for online discussion. As research suggests asynchronous online discussion can be unsatisfying and unproductive (i.e., Aloni and Harrington 2018), our two studies jointly indicate that students find SA satisfying and can use it as a productive means to construct knowledge.

There are two limitations of our study design that other researchers of SA and collaborative learning should work to mitigate. Both concern the extent to which we were able to comprehensively document students’ discursive activities in context, a methodological challenge noted in the CSCL literature that reflects complex social, cultural, and cognitive qualities of group discourse as situated across meaning-making contexts (i.e., Arvaja 2011). First, despite frequent coordination with participating faculty we had limited access to on-the-ground and online learning environments. Accordingly, we were unable to document via direct observation how SA was introduced to students as an approach to online discussion, nor were we able to observe how SA activities were orchestrated in coordination with other synchronous course activities (i.e., Zhu, Shui, and Chen 2020). A second limitation concerned unanticipated technical challenges that constrained our ability to document with nuance the online discursive context within which KC activities occurred. While Hypothesis SA has been extensively studied across various open and group-based online learning arrangements (i.e., Allred, Hochstetler, and Goering 2020; Kalir 2020a; Goller *et al*. 2021), the use of this technology within a university LMS is a recent development. Unexpected difficulties in mapping annotations to readings, caused by time-bound URLs given to students, curtailed our efforts to trace how KC activities progressed through a given text. We recommend future SA studies of Hypothesis establish technical workflows to contextualize discussion at multiple scales (i.e., thread, text, course), and examine threads as a unit of analysis to better understand the social sense-making processes of students as KC activities occur over time (i.e., Eryilmaz *et al*. 2013).

In light of our study’s strengths and limitations, this case described undergraduate student participation in asynchronous, group-based online discussion by detailing the extent to which their SA was discursive, and by analyzing the presence and prevalence of KC activities in SA. Notably, only a quarter (24%) of student SA across all three courses was discursive, although this varied substantially between courses. Threads accounted for almost half the SA written in GERO, over a third of the SA in GSWS, and less than one-tenth of the SA in PUB (annotations in PUB comprised more than half the total corpus, see Table 3). Nonetheless, online discussion did evidence all seven KC activities when students’ SA was discursive, albeit to differing degrees. Across the annotation corpus, interpretation was by far the most common KC activity ( Figure 2), a finding consistent with previous research ( Eryilmaz *et al*. 2013; Plevinski *et al*. 2017). Yet unlike Eryilmaz *et al.’* (2013) analysis of KC sequences within SA threads, we found that interpretation seldom elicited peer response ( Figure 4).

Our exploratory study is pertinent to long-standing interest in the “social life” of texts ( Brown and Duguid 1996) and the value of collaborative annotation (i.e., van der Pol, Admiraal, and Simons 2006) during asynchronous online discussion. Moreover, our findings about KC activities may be useful for researchers interested in the cognitive and social qualities of student annotation. For example, with respect to Gao *et al.’* (2013) model of productive online discussion, we found student participation in SA primarily demonstrated discussion for comprehension (as evidenced by the KC activities of interpretation and elaboration) as well as discussion for improved understanding. Yet our analysis of the distribution of KC activities by course and category ( Figure 3) revealed that students less frequently utilized SA to critique or actively negotiate meanings, reconsider assertions, or revise their thinking. KC activities analogous to engagement with diverse perspectives—like elaboration, questioning, consensus building, and conflict—were identified within discursive SA though in varying degrees ( Table 4). We found noteworthy course-level variation in how students wrote and shared annotation as a participatory social process through which divergent ideas were subsequently negotiated and synthesized (i.e., Onrubia and Engel 2009). In this respect, we speculate that discursive SA productively mediated the ongoing and social negotiation of meaning-making in GERO, perhaps also in GSWS, though probably not in PUB where only 6% of course SA were discursive. SA can, in some circumstances, make visible complex group-level cognitive processes (like conflict and consensus building) through online discourse. Moreover, our prior research indicated that students perceived social value in reading and writing annotation to clarify confusion, confirm ideas, and engage diverse perspectives ( Kalir *et al*. 2020). Nonetheless, further research should clarify the processual ways in which discursive SA aids student groups in sharing conflicting ideas and synthesizing among divergent perspectives.

Notably, student participation in SA may also reflect course-specific factors, including instructor expectations about online discussion. In PUB—the largest course in our sample—students were required to write at least two annotations per reading, perhaps explaining why every student in the class annotated and wrote annotations of the greatest length ( Table 1). However, the PUB assessment rubric did not emphasize peer interaction, which may explain, in part, both the overwhelming quantity of no-thread SA (94% of the course annotation) and interpretation as that course’s prominent KC activity ( Figure 3). In GERO, alternatively, an assessment rubric incentivized discursive SA. Students in this course were evaluated, partly, on “engaging with other students (responding to others’ comments).” It may not be surprising that GERO featured the highest percentage of top-of-thread SA and peer replies among courses. This may also explain other characteristics of GERO annotations, such as the relatively high prevalence of consensus building, and the fact that less frequent KC activities—like consensus building, support, and conflict—comprised over one-third of course SA (the highest percentage of such KC activities in the study). Across all courses, instructors approached their involvement in SA from a “more is not always better” ( Zhu *et al*. 2020, 267) stance, collectively writing just 1% of the corpus. Given difference and similarity among instructional contexts and practices, future SA research—and, in particular, design-oriented rather than descriptive studies—should carefully consider how to scaffold students’ writing of SA so that it is both discursive and encouraging of particular KC activities.

This study provides further evidence that SA is a productive form of online learning enabling students’ collaborative KC. As such, we conclude our discussion by noting methodological and instructional implications that should be useful for other researchers and educators interested in SA. First, with only a handful of SA studies examining KC activity, we made an intentional decision to borrow Plevinski *et al.’* (2017) codebook rather than create a bespoke analytic scheme. We encourage other SA researchers to take up this common method when studying KC in online learning with larger samples of students, more courses, and other disciplines. Second, future studies might also combine thread-level analysis of student annotation with additional data sources like educator interviews, student focus groups, and direct course observations (as with the methods in Schneider *et al*. 2016) to better contextualize how SA enables KC progressions within the broader social life of a course. Third, a range of instructional opportunities are also possible if instructors can, in real-time, be made aware of students’ SA use, emerging participation patterns, and the ongoing development of KC activities. The design of complementary technologies, such as learning analytics dashboards that dynamically report student SA ( Kalir 2020a), could be attuned to KC patterns so that instructors might better support group-level discourse across course texts and orchestrate sequential collaborative activities. Aiding instructor knowledge of student SA through efficient feedback processes would also help inform the ways in which instructors participate in online discussion to clarify student misunderstanding and build connections to relevant disciplinary literature and methods. New instructional methods could also encourage students to write SA evidencing a wider range of KC activities. Our final instructional recommendation is that instructors model and transparently assess how SA enables productive, discipline-specific online discussion in accordance with course learning objectives ( Zhu, Shui, and Chen 2020).

## Conclusions

This study examined the affordances of SA as asynchronous online discussion and analyzed how SA enabled students’ KC activities. Our case provides further evidence that SA, as mediated by the Hypothesis technology, is a productive form of online discussion through which undergraduate students in multiple disciplinary contexts interacted with peers (i.e., Kalir *et al*. 2020), made sense of academic content (i.e., Kararo and McCartney 2019), and constructed knowledge by reading and writing together (i.e., Sprouse 2018). As the first descriptive cross-disciplinary account of students using Hypothesis SA to construct knowledge together, this case details the ways in which SA made cognition visible and collaborative activity possible (i.e., Kalir 2020a; Chan and Pow 2020). Undergraduate student SA as online discussion was a socio-cognitive context within which we identified the predominance of textual interpretation, a supporting set of KC activities that included elaboration, clarification, and asking questions, as well as a less frequent group of KC activities that included consensus building, support, and conflict. Given long-standing interest in SA and learning (i.e., Novak *et al*. 2012) and recent changes in digital education, this study offers a valuable and timely contribution to the literature. While our broader study was designed and conducted prior to the coronavirus disease 2019 (COVID-19) pandemic, we analyzed data for this case and wrote this article throughout the disrupted 2020-21 academic year. Amid the pandemic, hybrid and online courses became the primary mode of learning in higher education. Changes to digital learning arrangements exacerbated by the pandemic may, in part, benefit from light-touch and highly-collaborative learning technologies—like SA—that promote online discussion and aid student KC about discipline-specific concepts, methods, and content. In this respect, our study extends an established line of inquiry about the educational benefits of SA, provides practical and relevant insight about SA as asynchronous online discussion, and can help shape future research about the social practices and cognitive qualities of collaborative learning in higher education.

## Data availability

### Underlying data

Dataverse: Knowledge Construction Activities in the Online Social Annotations of Three Classes, https://doi.org/10.7910/DVN/SVDFCF ( Morales, Fleerackers, Alperin 2022).

This project contains the following underlying data:

- AnnotationsCoded_Course.tab (raw coded knowledge construction activities)
- Codebook.pdf

Data are available under the terms of the Creative Commons Zero “No rights reserved” data waiver (CC0 1.0 Public domain dedication).
