# Supplementary material for: Using social annotation to construct knowledge with others: A case study across undergraduate courses
Source: F1000Res. 2022 Apr 1;11:235. Originally published 2022 Feb 25. [Version 2] doi: 10.12688/f1000research.109525.2 (PMC8938630; doi:10.12688/f1000research.109525.2)
Supplement: Peer review history

# Peer review: Reviewer response for version 2

**DOI:** 10.5256/f1000research.122901.r129588

**Published:** 2022-04-11

**Authors:** Federico Pianzola

**Affiliations:** 1 University of Groningen, Groningen, Netherlands Antilles

I do not have any new comments.

Is the case presented with sufficient detail to be useful for teaching or other practitioners?

Yes

Is the work clearly and accurately presented and does it cite the current literature?

Yes

If applicable, is the statistical analysis and its interpretation appropriate?

Not applicable

Are all the source data underlying the results available to ensure full reproducibility?

Partly

Are the conclusions drawn adequately supported by the results?

Yes

Is the background of the case’s history and progression described in sufficient detail?

Yes

Reviewer Expertise:

digital social reading, computational literary studies

I confirm that I have read this submission and believe that I have an appropriate level of expertise to confirm that it is of an acceptable scientific standard.

# Peer review: Reviewer response for version 1

**DOI:** 10.5256/f1000research.121034.r125503

**Published:** 2022-03-21

**Authors:** Federico Pianzola

**Affiliations:** 1 University of Groningen, Groningen, Netherlands Antilles

Morales *et al.* provide a robust investigation of social annotation in the context of higher education by looking at 3 different undergraduate courses from 3 different disciplines. The large number of annotations analysed suggests that the results may be valid in many other educational contexts. The contextualisation of the research within an existing scholarly tradition is very accurate and the choice of using an established protocol (codebook) for qualitative research is excellent for the comparability of the results.

 The discussion and interpretation are appropriate and rightfully acknowledge limitations that can occur in field studies and prevented the researchers to gain more detailed insight.

 An important issue that is not clear from the article is whether informed consent has been obtained from the students. Moreover, it is not possible to fully reproduce the results of the study because the full text of the annotations is missing.

 A few smaller remarks that could improve the presentation of the research:

- Given the inconsistencies between academic calendars in various countries and the differences between Northern and Southern hemisphere, it would be better to express the time range of the data collection in number of weeks and months of the year. Now it is only stated: winter 2019 semester.
- In place of Table 4, Figure 3 could be modified by using stacked bars to show the proportion of annotations for each course within each bar (e.g. 3 different shades of orange for no-thread)

Is the case presented with sufficient detail to be useful for teaching or other practitioners?

Yes

Is the work clearly and accurately presented and does it cite the current literature?

Yes

If applicable, is the statistical analysis and its interpretation appropriate?

Not applicable

Are all the source data underlying the results available to ensure full reproducibility?

Partly

Are the conclusions drawn adequately supported by the results?

Yes

Is the background of the case’s history and progression described in sufficient detail?

Yes

Reviewer Expertise:

digital social reading, computational literary studies

I confirm that I have read this submission and believe that I have an appropriate level of expertise to confirm that it is of an acceptable scientific standard.

# Response

**Published:** 2022-03-26

**Authors:** Juan Pablo Alperin

**Affiliations:** Simon Fraser University, Canada

Thank you for taking the time to provide us with this review. We have now uploaded a revised manuscript that addresses many of your comments and provide a response to all here.

 In his review, Dr. Pianzola expressed concern regarding whether informed consent was obtained from the students. We take research ethics seriously and sincerely appreciate his concern. The short answer is that we did not obtain consent because the data were collected for the assessment of students as part of their regular course work. We then followed the Canadian Tri-Council Policy Statement (as is required at Canadian Universities) and affirm our understanding that our study is exempt from ethics board review according to Article 2.5 of this policy. A statement to this effect appears at the beginning of the Methods section.

 Dr. Pianzola also noted that different academic calendars, especially between the Northern and Southern hemispheres, would not be clear of when "Winter Semester" has taken hold. Again, we deeply appreciate this small, but significant suggestion. This was an oversight on our part, as we do attempt to avoid centering the Global North in so much of our other work. We have now changed this in the abstract and in the text to indicate it was between January and April 2019.

 Finally, Dr. Pianzola suggests that the results presented in Table 4 could be incorporated into Figure 3. We have not done this for two reasons: 1) we believe figures can be less effective if they present too much information and become confusing or overwhelming for readers; but more importantly, 2) it would be difficult to read from the stacked-bar graph suggested the information being highlighted by Table 4.

# Peer review: Reviewer response for version 1

**DOI:** 10.5256/f1000research.121034.r125500

**Published:** 2022-03-17

**Authors:** Emily J. Ragan

**Affiliations:** 1 Metropolitan State University of Denver, Denver, CO, USA

Morales *et al.* explore knowledge construction and its intersection with social annotation, where students collaboratively annotate a text. They analyzed a full term worth of student annotations from three different upper division undergraduate courses, comparing the number of annotations that stood alone (no-thread) to those that became part of a thread (with one or more student(s) responding to an initial post). They also coded student annotations of course readings based on seven established types of knowledge construction and compared patterns across the three courses. Then, combining the first two analyses, they looked at how knowledge construction activities related to no-thread posts or discourses (a thread) between students. Student survey data on student perceptions of social annotation from same three courses has been shared in a previous paper (Kalir *et al.* 2020).

 The percent of annotations that became threads varied between courses, from 6% to 46%. A vast majority of annotations (94%) were found to be related to at least one of the seven knowledge construction categories. The most common categories across all three courses were interpretation and elaboration. Certain types of knowledge construction were much more likely to occur in responses to threads: consensus building, support, and conflict. This paper offers a strategy for cataloging the knowledge construction activities within social annotation, which may be helpful for faculty seeking to better understand how their students are using social annotation. It also offers a way faculty could measure potential impacts of changes to assignment instructions and/or assessment rubrics on student knowledge construction activities as documented through social annotation.

**Introduction**

 I recommend the authors acknowledge that knowledge creation can be individual as well as social. In the section “Knowledge construction” they state: With roots in cognitive psychology and constructivism, KC is defined in the CSCL literature as a social process "by which students solve problems and construct understanding of concepts, phenomena, and situations" ( van Aalst 2009, 261). However, I note that van Aalst’s next sentence is “It is effortful, situated, and reflective, and can be individual or social (Sullivan Palincsar 1998).” I think it is a mistake to imply that all knowledge construction is inherently social. Social annotation allows individual effort and reflection to be made visible to the group, thus blurring the boundary between individual and social, but the fact that individuals construct knowledge cannot be ignored.

 A brief introduction of coordination activities in the section “SA enabling KC” would be helpful. I also personally would prefer to have social annotation and knowledge construction written out, avoiding SA and KC abbreviations.

**Methods**

 The format that I assume is indicating a specific annotation in the last sentence of the Methods, [631] and [739], is slightly different that that used in other portions of the paper, where the number in square brackets is preceded by SA.

 No statistical analyses were performed. While I believe that is reasonable for the scope of this descriptive project, future work on this topic would be strengthened by greater statistical rigor.

**Discussion**

 There is a sentence in the discussion that I found unclear. “Unexpected difficulties in mapping annotations to readings, caused by time-bound URLs given to students, curtailed our efforts to trace how KC activities progressed through a given text.” I assume that the Hypothesis API, which provided detail about annotations, lacked information relating threads to specific readings. Clearly stating what you would have liked to do (compare knowledge creation activities across different texts, or within different sections of the same text, etc.) would help the reader better understand this specific limitation. More detail in the Methods section about the annotation data and metadata available from the Hypothesis API may also be helpful.

**Data availability**

 The data after coding into knowledge construction categories is available but lacks the original annotation text.

Is the case presented with sufficient detail to be useful for teaching or other practitioners?

Yes

Is the work clearly and accurately presented and does it cite the current literature?

Yes

If applicable, is the statistical analysis and its interpretation appropriate?

Not applicable

Are all the source data underlying the results available to ensure full reproducibility?

Yes

Are the conclusions drawn adequately supported by the results?

Yes

Is the background of the case’s history and progression described in sufficient detail?

Yes

Reviewer Expertise:

Science education, biochemistry

I confirm that I have read this submission and believe that I have an appropriate level of expertise to confirm that it is of an acceptable scientific standard.

## References

[1] : Social constructivist perspectives on teaching and learning. *Annu Rev Psychol* .1998;49: 10.1146/annurev.psych.49.1.345 345-75 10.1146/annurev.psych.49.1.345 15012472

[2] : Social annotation enabling collaboration for open learning. *Distance Education* .2020;41(2) : 10.1080/01587919.2020.1757413 245-260 10.1080/01587919.2020.1757413

[3] : Distinguishing knowledge-sharing, knowledge-construction, and knowledge-creation discourses. *Int J Comput Support Collab Learn* .2009;4(3) : 10.1007/s11412-009-9069-5 259-287 10.1007/s11412-009-9069-5 32214914PMC7088907

# Response

**Published:** 2022-03-26

**Authors:** Juan Pablo Alperin

**Affiliations:** Simon Fraser University, Canada

Thank you for taking the time to provide us with this thoughtful review. We are uploading a revised manuscript that addresses these comments in addition to this response.

**Introduction**

 Dr. Ragan helpful suggests that we "acknowledge that knowledge creation can be individual as well as social". We very much agree with this suggestion and appreciate the opportunity to better clarify KC (in general) and the particular stance toward KC that we are extending in our paper. We have revised the "Knowledge Construction" subsection to better reflect that KC is both an individual and social phenomena, and that we are highlighting the social qualities and affordances of KC within our study (and related CSCL literature). The new text can be found in the first few sentences of this section.

 Dr. Ragan also suggests that we add a "brief introduction of coordination activities in the section 'SA enabling KC'". We have now edited the opening paragraph of this section to do this, including the addition of a new second sentence in this section.

 Finally, in the introduction, Dr. Ragan suggests we avoid using the SA and KC abbreviations. We discussed this, but feel that the terms too frequent in the manuscript and that writing them out would only encumber the reader. We see this as a matter of style and have opted to retain the abbreviations.

**Methods**

 We have now standardized the way in which we identify participants by adding "SA" inside the square brackets. Thank you for catching this discrepancy.

**Discussion**

 Dr. Ragan noted a sentence that was unclear. We agree that it was so and have re-written the sentence. The new sentence can be found near the end of the third paragraph of the Discussion.

**Data Availability**

 Both reviewers noted that we did not publish the text of the annotation with our open dataset. This was intentional, as we did not feel like we could sufficiently anonymize the texts to guarantee the privacy of the students and the instructors.
